# Observation of Electrostatically Driven Surface Adsorption in Mixed Surfactant Systems

**DOI:** 10.1021/acs.jpclett.3c03377

**Published:** 2024-02-02

**Authors:** Aswathi Vilangottunjalil, Jan Versluis, Huib J. Bakker

**Affiliations:** AMOLF, Ultrafast Spectroscopy, Science Park 104, 1098 XG Amsterdam, Netherlands

## Abstract

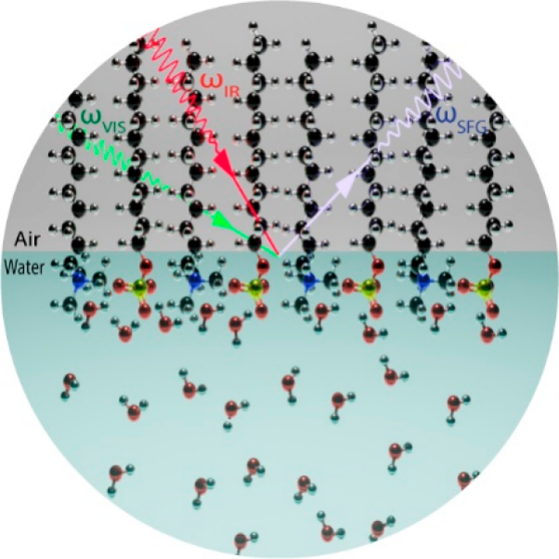

We employed heterodyne-detected vibrational sum-frequency generation (HD-VSFG) spectroscopy to obtain a molecular-level understanding of the interaction between the anionic surfactant sodium dodecyl ammonium sulfate (SDS) and the cationic surfactant dodecyltrimethylammonium bromide (DTAB). We observed that these surfactants show a strong cooperative effect on their adsorption to the water–air interface. Even at bulk concentrations 1000 times lower than the critical micelle concentrations of SDS and DTAB, a nearly complete surface surfactant layer is observed when both surfactants are present. This strong enhancement of the surface concentrations of DS^–^ and DTA^+^ can be quantitatively explained from the favorable Coulomb interaction of the oppositely charged headgroups of DS^–^ and DTA^+^ and the electrostatic interactions with their counterions. The HD-VSFG results are complemented by a modified Langmuir adsorption model in which we include the free energy associated with the electrostatic interactions of the surfactant ions and their counterions.

The unique ability of surfactants to lower the surface tension and form organized structures at interfaces makes them indispensable in various applications, including agriculture,^1,2^ the textile industry,^3^ and environmental chemistry.^4−8^ Surfactants also contribute to various cellular functions and protein stability.^9^ In medicine they are utilized in drug delivery^10−12^ and pharmaceutical formulations.^13^

The combination of different surfactants can lead to quite interesting synergistic effects.^14^ Recently, we studied binary mixtures of the anionic surfactant sodium dodecyl sulfate (SDS) and the nonionic hexaethylene glycol monododecyl ether (C_12_E_6_). We found that the effect of SDS on the orientation of interfacial water molecules is multifold enhanced in the presence of C_12_E_6_ at a critical micellar concentration (CMC) of 70 μM.^15^ Catanionic surfactant systems, which combine cationic and anionic surfactants, also show strong synergistic effects, resulting in a reduced CMC and enhanced surface activities,^16^ which in turn leads to large changes in solubility and degree of micellar aggregation.^17^ For instance, Nguyen and co-workers demonstrated that a mixture of the anionic surfactant SDS and the cationic surfactant dodecyl amine hydrochloride (DAH) shows a strong cooperative effect in the adsorption and packing of the surfactants at the water–air interface.^18^ Kumar and co-workers studied the catanionic system of SDS and cetyltrimethylammonium bromide (CTAB) at the water surface^19^ with intensity vibrational sum-frequency generation (VSFG) measurements. They found that for a 1:1 mixture of SDS and CTAB, the OH stretching modes of the water molecules show no response, indicating that the interfacial water molecules do not possess a net orientation.

In this paper we report on a study of the interaction of anionic SDS and the cationic surfactant dodecyltrimethylammonium bromide (DTAB) with heterodyne detected vibrational sum-frequency generation (HD-VSFG) spectroscopy.^20,21^ This technique has significantly advanced our understanding of interfaces at the molecular level.^22,23^ The details of the experimental setup can be found in the Supporting Information. SDS (CMC = 8.3 mM^24^) and DTAB (CMC = 14.6 mM^25^) are two oppositely charged ionic surfactants with the same alkyl chain length. We varied the concentrations of SDS and DTAB from 10 to 200 μM. We observed a strong cooperative absorption of both surfactants at the water–air interface and monolayer formation at concentrations that are orders of magnitude lower than the CMC’s of the two surfactants. Furthermore, we developed a modified Langmuir adsorption model in which we include the electrostatic energy of the surfactant ions and their counterions.

In Figure 1, the Im[χ^(2)^] spectra of neat water, an aqueous solution of 50 μM SDS, and an aqueous solution of 50 μM DTAB are displayed in the frequency range between 2800 and 3600 cm^–1^, all measured in SSP^20^ polarization configuration. The HD-VSFG spectrum of water shows a weak broad negative band between 3200 and 3600 cm^–1^ that is assigned to the OH stretching frequencies of hydrogen-bonded water molecules^26^ (Figure 1, cyan). In the presence of charged surfactants, the signal of the OH vibrations is strongly enhanced, and the sign of the signal depends on the charge of the surfactant.

**Figure 1:**
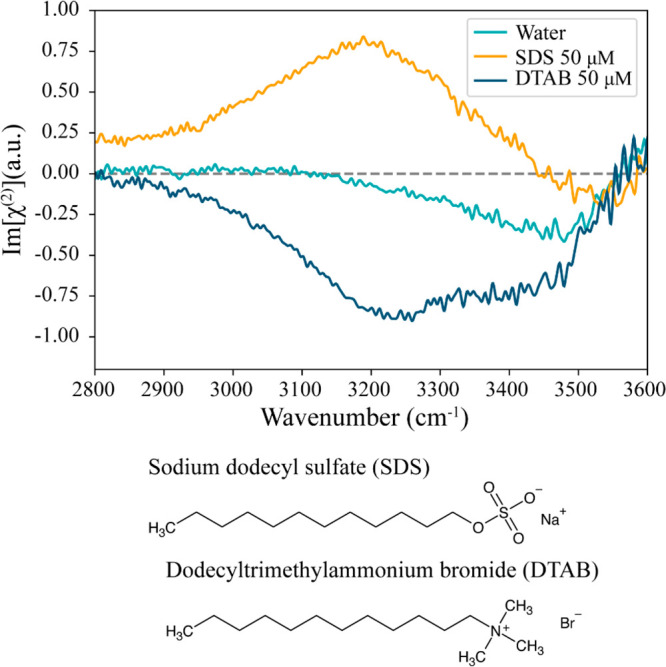
Im [χ^(2)^] spectra of neat water (cyan) and aqueous solutions of 50 μM anionic surfactant sodium dodecyl sulfate (SDS) (orange) and 50 μM cationic surfactant dodecyltrimethylammonium bromide (DTAB) (dark blue). The molecular structures of SDS (left) and DTAB (right) are shown below the spectra.

The sign of the Im[χ^(2)^] spectrum of the OH vibrations reflects the orientation of its vibrational transition dipole moment with respect to the surface.^27^ A negative sign of the OH stretch band (Figure 1, blue) indicates an orientation of water molecules at the interface, with their hydrogen atoms pointing toward the bulk. This is observed for solutions with positively charged DTA^+^ ions at the surface.^28^ In the spectrum of the solution with SDS a strong positive signal is observed (Figure 1, orange). In this case the water molecules have a net orientation with their hydrogen atoms pointing toward the negative charges of the DS^–^ ions at the surface.^28^

The Im[χ^(2)^] spectra of SDS and DTAB shown in Figure 1 do not show any response of the CH stretches of the methyl groups of the alkyl chains of the surfactants, which indicate a quite low surface coverage of surfactant ions. Here, it should be realized that the spectra are measured for solutions with a relatively low surfactant concentration of 50 μM. For solutions containing only SDS or only DTAB the response of the CH vibrations is usually observed for surfactant concentrations approaching the CMC, i.e., in the millimolar region.^29^

Figure 2 shows HD-VSFG spectra of solutions containing both SDS and DTAB in a concentration ratio of 1:1. We observe two negative features at 2878 and 2939 cm^–1^, a sharp positive peak at 2965 cm^–1^, and a broad positive peak at around 3200 cm^–1^. The 2878 cm^–1^ is assigned to the symmetric stretch vibration of the terminal methyl group, and the 2939 cm^–1^ is assigned to the Fermi resonance of the symmetric CH stretch vibrations and the overtone of the CH bending mode of the methyl group.^28,30^ The small positive peak at 2965 cm^–1^ is assigned to the antisymmetric CH stretch vibration of the terminal methyl group.^15^ This observation of these CH signals in the binary surfactant mixture suggests strong orientational ordering of the hydrophobic tails of the surfactants. Barely any signal from the symmetric stretch of the methylene group around 2850 cm^–1^ is detected, which indicates that the tails are quite well aligned in an all-trans (zigzag) conformation.^31^

**Figure 2:**
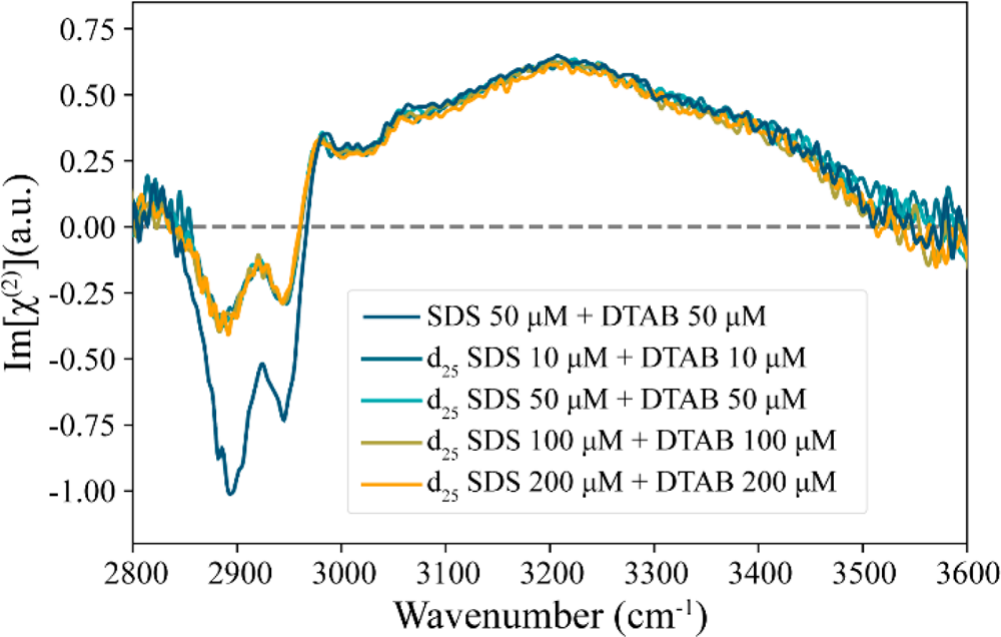
Im[χ^(2)^] spectra of aqueous solution of several 1:1 ratios of mixed surfactants of deuterated SDS and DTAB between 10 and 200 μM at the water–air interface. For comparison, the 1:1 ratio of 50 μM nondeuterated SDS and DTAB.

To distinguish the individual contributions of the SDS and DTAB alkyl tails to the CH bands in the HD-VSFG spectrum, we also measured the HD-VSFG spectrum of a 1:1 solution of deuterated 50 μM d_25_ SDS and 50 μM DTAB (Figure 2 (cyan)). For perdeuterated SDS, the 2800–3000 cm^–1^ response of the CH stretches is changed to a response of CD stretch vibrations at significantly lower frequencies of 2000–2200 cm^–1^.^30^ We find that this isotopic exchange reduces the CH signal amplitude by half, which indicates the presence of nearly equal densities of DS^–^ and DTA^+^ at the surface.

As a next step, we varied the concentration of DTAB from 1 to 200 μM, while keeping the concentration of SDS at 50 μM (Figure 3). We observe that the addition of a small amount of 10 μm DTAB to 50 μM d_25_ SDS leads to the appearance of strong CH signals and a small increase of the positive Im[χ^(2)^] response of the water OH stretch vibrations. A further increase of the DTAB concentration up to 200 μM leads to a decrease of the water OH signal, while the CH signal remains almost unchanged.

**Figure 3:**
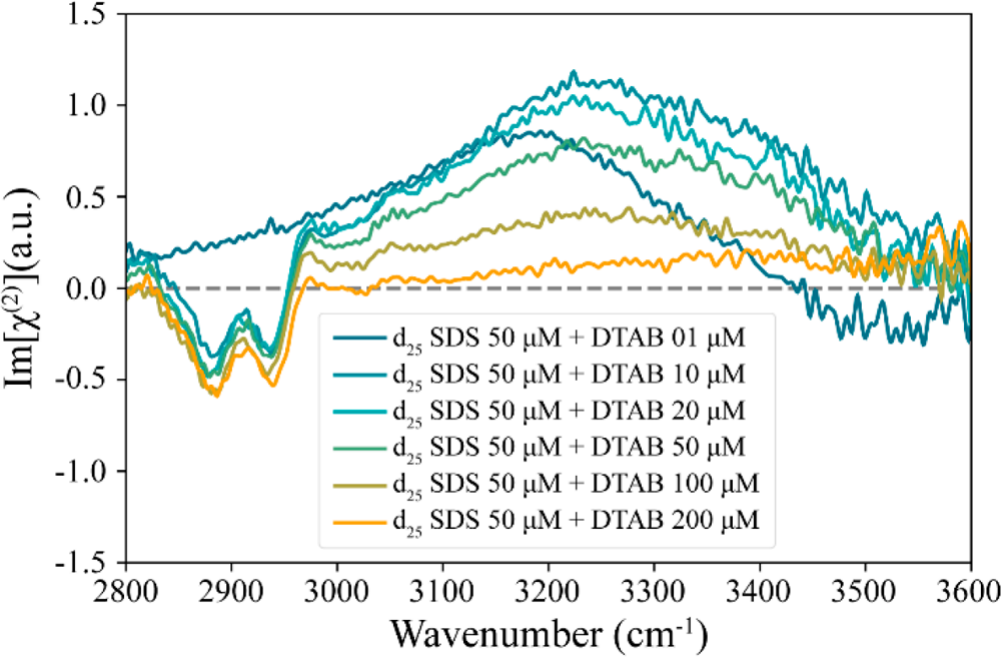
Im[χ^(2)^] spectra of aqueous solution of surfactant mixtures of 50 μM deuterated SDS with different concentrations of DTAB ranging from 1 to 200 μM at the water–air interface.

The appearance of the CH signals upon addition of 10 μM DTAB to a solution of 50 μM SDS indicates a strong increase in surface density of the surfactants. The favorable Coulomb interaction between the oppositely charged headgroups of DS^–^ and DTA^+^ enables both surfactants to come to the surface, leading to a surface that is considerably covered with approximately equal amounts of DS^–^ and DTA^+^. The increase in surface density of DS^–^ and DTA^+^ also allows the surfactant ions to pack more closely, leading to a better ordering of the alkyl tails of the surfactant ions, which further enhances the CH signals.

To analyze the Im[χ^(2)^] spectra quantitatively, we decompose the responses measured at different DTAB concentrations by fitting the spectra to a set of Lorentzian bands. The spectra in Figure 3 are decomposed into four Lorentzian bands centered at 2878, 2939, 2965, and 3248 cm^–1^, describing the symmetric CH_3_ stretch vibration of the methyl group, the Fermi resonance of this vibration with the overtone of the CH_3_ bending mode, the antisymmetric CH_3_ stretch vibration of the methyl group, and the OH stretch vibrations of the water molecules, respectively. In the fitting, we kept the widths of all Lorentzian bands the same at all DTAB concentrations, and only allowed the amplitudes of the bands to change in dependence on the DTAB concentration. A more detailed explanation of the fitting procedure can be found elsewhere.^42^ The amplitudes resulting from the fit are shown in Figure 4. A selection of fitted spectra is shown in the Supporting Information.

**Figure 4:**
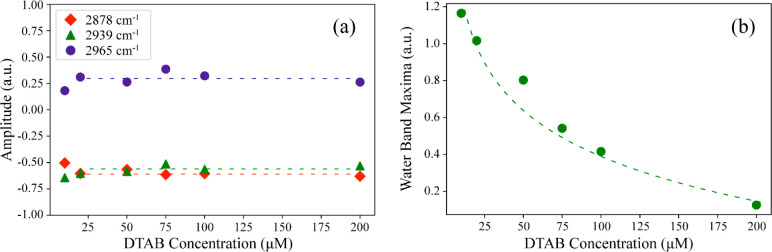
(a) Amplitudes of the CH stretches vibrational bands centered at 2880, 2937, and 2970 cm^–1^, extracted from the fitting procedure as a function of concentration of DTAB. (b) Amplitude of the water OH stretch signal extracted from the fitting procedure as a function of concentration of DTAB. The dashed lines are guides to eyes.

The amplitudes of the methyl stretch vibrational modes extracted from the fitting procedure (Figure 4a) hardly change with increasing DTAB concentration, which implies that increasing the bulk concentration of DTAB from 10 to 200 μM does not lead to a significant change in the surface densities of these surfactants. This near-complete absence of change of the surface density of DTA^+^ depending on its bulk concentration is a clear deviation from the Langmuir adsorption isotherm model^32^ which predicts that in a binary surfactant mixture, the surfactants compete for the surface area. We also performed HD-VSFG measurements in the CD stretching region of the deuterated SDS for these solutions and found that the amplitudes of the CD stretching vibrational bands also remained the same throughout the concentration series (see Figure S3). We thus find that even a 20-fold increase in bulk DTAB concentration does not significantly increase the surface density of either of the surfactants, which implies that already for a solution of 10 μM SDS and 10 μM DTAB the surface is quite well covered with a layer of DS^–^ and DTA^+^ surfactant ions in a concentration ratio of 1:1.

## Calculation of the Surface Coverage with a Modified Langmuir Adsorption Model

To explain the dependence of the amplitudes of the CH stretch and OH stretch HD-VSFG signals on the bulk concentrations of SDS and DTAB, we performed model calculations in which we extended the standard Langmuir adsorption isotherm. One assumption of the standard Langmuir model is that the surfactant molecules do not interact with each other, which is not valid in the present case. The surface occupancy obtained with the Langmuir adsorption model is
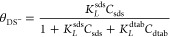
1
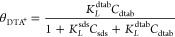
2The *K*_*L*_ terms represent Langmuir equilibrium adsorption constants which can be expressed in terms of Gibbs free energy,^33^ and the *C* terms represent the bulk concentrations.
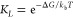
3where Δ*G* is the total Gibbs free energy change associated with adsorption to the surface, *k*_b_ is Boltzmann’s constant, and *T* is the temperature. For the case of a charged surfactant like DS^–^ and DTA^+^, the values of Δ*G* and thus *K*_*L*_ depend on electrostatic interactions governed by the ionic strength and the presence of other charged surfactants and their counterions. Δ*G* can thus be separated in a nonelectrostatic part Δ*G*_nel_ which accounts for the standard Gibbs free energy of adsorption and an electrostatic part Δ*G*_el_:
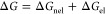
4The electrostatic energy term is composed of the energy associated with adsorption of the surfactant ions at the surface and that of their counterions in the diffuse double layer.
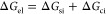
5The counterions are distributed over a region that extends some distance from the surface into the bulk because of the thermal motion of counterions. Hence, the energy associated with the counterions can be determined by integrating over the depth of the double layer, as shown in eq 6.
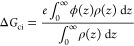
6where ρ(*z*) is the charge density and ϕ(*z*) is the electrostatic potential at position *z*. The electrostatic energy of the surfactant ions at the surface is −*e*ϕ_0_. Solving the Poisson–Boltzmann equation for the potential ϕ(*z*), using the low potential assumption,^33^ we obtain for Δ*G*_el_
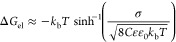
7where σ is the surface charge density that depends on the surface occupancy and the area *a* occupied by each surfactant obtained from the Langmuir isotherm (σ = *e*(θ_DTA^+^_ – θ_DS^–^_)/*a*), *C* is the total concentration of monovalent salts (*C*_sds_*+ C*_dtab_) in mol/L, and ε is the static permittivity of the solution.

We solve eqs 1, 2, 3, 4, and 7 self-consistently for θ_DTA^+^_ and θ_DS^–^_ for each mixture of concentrations *C*_sds_ and *C*_dtab_. For monovalent electrolytes, the interfacial potential^34^ ϕ(0) can be related to the surface charge density by the Grahame equation.^35,36^

**Figure 5:**
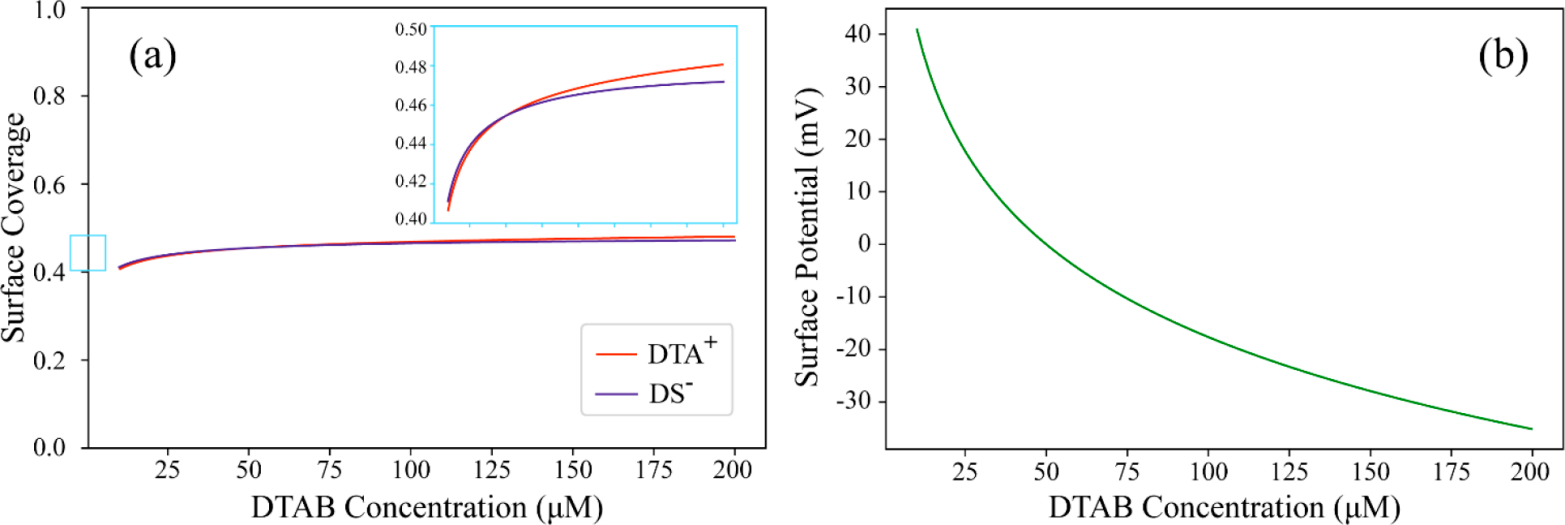
(a) Surface occupancy of DTA^+^ and DS^–^ as a function of the concentration of DTAB in the presence of 50 μM SDS, calculated by using the model described in the text. The inset shows the same graph with a zoomed-in vertical scale. (b) Surface potential expressed in mV, calculated from the Grahame equation (see text).

In Figure 5a, we show the calculated surface occupancies of DS^–^ and DTA^+^ for solutions with an SDS concentration of 50 μM and different DTAB concentrations. We find that the surface occupancy of both surfactants remains almost the same with increasing DTAB bulk concentration, which agrees well with our experimental data (Figure 4a). This contrasts the standard Langmuir adsorption isotherm model that predicts that with increasing DTAB bulk concentration, the surface occupancy of SDS strongly decreases, while that of DTAB strongly increases (Figure S4). By including the free energy associated with the electrostatic interaction, we see that the surface has a strong tendency to maintain a state of neutrality, i.e., to have equal amounts of DS^–^ and DTA^+^ at the surface irrespective of their bulk concentrations.

Despite the strong tendency to maintain electric neutrality, the surface concentrations of DS^–^ and DTA^+^ do show a weak dependence on the bulk concentrations, as shown in the inset of Figure 5a. When the bulk concentration of DTAB is increased from 10 to 200 μM, there is a transition from a slightly higher DS^–^ concentration to a slightly higher DTA^+^ concentration at the surface. The differences are maximally 2%, meaning that these variations do not lead to a significant increase in the CH signals of DTA^+^.

## Water Signal and Surface Potential

In contrast to the CH signals, the Im[χ^(2)^] response of the water OH stretch vibrations does show a strong dependence on the added DTAB concentration, as shown in Figures 3 and 4b. The sign and amplitude of the water signal are determined by the net orientation of the water molecules in the layers close to the surface, and this orientation is largely determined by the sign and strength of the electric field exerted by the surface. This electric field is directly related to the surface potential that in turn is governed by the difference in surface concentration of DS^–^ and DTA^+^.

In Figure 5b, we show the surface potential as a function of DTAB concentration, calculated with the modified Langmuir adsorption model employing the Grahame equation. We find that the surface potential decreases from 41 to −35 mV when the DTAB concentration is increased from 10 to 200 μM. For a DTAB concentration of 50 μM, the surface potential equals zero. The functional dependence of the amplitude of the water signal shown in Figure 4b on the DTAB concentration corresponds quite well with the functional dependence of the surface potential on the DTAB concentration but with a clear vertical offset.

When the bulk concentrations of SDS and DTAB are the same, the surface concentrations of DS^–^ and DTA^+^ are also nearly the same and the surface potential vanishes, which implies that there is no surface electric field that orients the water molecules in the diffuse layer. In a previous intensity SFG study of mixtures of SDS and CTAB (which is related to DTAB, but with a longer alkyl chain), the OH signal indeed almost vanished for a 1:1 molar ratio of CTAB and SDS. In the present HD-VSFG study of mixtures of SDS and DTAB we do observe a clear positive Im[χ^(2)^] response of the water OH stretch vibrations for all solutions with a 1:1 ratio of SDS (or d_25_ SDS) and DTAB (Figure 2). This residual positive Im[χ^(2)^] water response likely originates from water molecules that are not in the diffuse layer but directly interacting with the headgroups of the DS^–^ and DTA^+^ ions. The negatively charged sulfate headgroup of DS^–^ will likely have a stronger orienting effect on nearby water molecules than the positively charged trimethylammonium headgroup of DTA^+^ because for the latter, the interaction with the positive charge is weakened due to the presence of intervening methyl groups. To test this hypothesis, we performed an experiment with dodecylammonium bromide (DAB) instead of DTAB (Figure S2). For DAB the interaction of the water molecules with the positive ammonium headgroup of DA^+^ will not be hindered by intervening methyl groups. Indeed we observe that the Im[χ^(2)^] water response completely vanishes for a solution of 1:1 DAB and SDS. Hence, the sulfate headgroup of DS^–^ indeed appears to have a more strongly orienting effect on the water OH groups than the trimethylammonium headgroup of DTA^+^.

A striking observation in Figures 2 and 3 is the emergence of strong CH vibrational signals for mixed SDS and DTAB surfactant solutions at concentrations that are 100–1000 times lower than the critical micelle concentrations of the separate surfactants. This finding shows that the surface density of a charged surfactant increases strongly if the solution additionally contains a low concentration of a surfactant of oppositely charge. This finding agrees with the results of a study of Bhattarai et al.,^25^ who evaluated the surface and thermodynamic properties along with the CMC of mixed surfactant systems of SDS and DTAB. From the surface tension measurements, they found that the CMC of SDS-rich and DTAB-rich mixtures is much lower than that of individual surfactants, indicating a strong synergistic effect for the surfactants to come to the surface. This effect also results in enhanced foamability^37^ and enhanced surface activities.^16^

The strong increase in the surface density of mixed solutions of surfactants of opposite charge can be well explained from the Coulomb attraction of the oppositely charged headgroups of the surfactants. According to the modified Langmuir model, the lowest free energy of adsorption to the surface is attained when the surface potential equals zero, corresponding to a 1:1 molar ratio of the oppositely charged surfactants at the surface. The results displayed in Figures 3 and 4a show that the amplitudes of the CH stretch vibrational bands hardly change with DTAB concentration, which implies that the molar ratio of DS^–^ and DTA^+^ in the surface layer is always approximately 1:1, even at a bulk concentration ratio of DTAB to SDS of 1:20. A similar observation was reported by Kawai et al. on mixed ionic surfactant solutions of SDS and cetylpyridinium chloride (CPC) using infrared external reflection (IER) spectroscopy.^38^ It was found that at all studied solution mole fractions of SDS and CPC, the monolayer at the solution–air interface had a 1:1 composition and that the alkyl chains of the surfactant molecules possess an all-trans conformation within the adsorbed monolayer.

We thus find that the surface density of a charged surfactant to the surface can be strongly increased by adding a surfactant of opposite charge. A similar effect can result from the addition of ordinary positive and negative ions to the solution, as the ions with an opposite charge to the surfactant can accumulate at the surface, leading to a favorable Coulomb interaction similar to that observed for surfactants of opposite charge. The added ions thus partly screen the Coulomb repulsion of the surfactant headgroups. In a recent study it was indeed found that the addition of NaCl to a solution of SDS leads to a very strong increase of the surface density of DS^–^ due to screening of the Coulomb repulsion between the DS^–^ headgroups in the plane of the surface by the added Na^+^ ions.^39^ This screening effect also explains why the effective CMC of charged surfactants decreases upon the addition of salts.^40,41^

The strong increase in surface density of DS^–^ by adding NaCl required concentrations of 10–100 mM of NaCl. Here, we observe that the addition of only 10 μM DTAB, i.e., a 1000 times lower concentration, to a solution of 50 μM SDS, already suffices to create a complete surfactant monolayer, as evidenced by the CH signals shown in Figure 3. The positively charged surfactant DTA^+^ is thus much more effective in screening the Coulomb repulsion between the negatively charged sulfate headgroups of DS^–^ ions at the surface than Na^+^ ions, which is a direct consequence of the much higher surface propensity of DTA^+^ compared to Na^+^.

The addition of a nonionic surfactant like C_12_E_6_ to a solution of SDS can also lead to an enhancement of the surface density of DS^–^.^15^ Obviously, a neutral surfactant does not screen the Coulomb repulsion between the DS^–^ headgroups, which implies that the enhancement of the DS^–^ surface density has a different origin. In this case, the enhancement results from the favorable van der Waals interaction of the alkyl tails of the C_12_E_6_ and DS^–^ surfactants which is in fact also present in the case of DTA^+^ and DS^–^. However, in the case of DTA^+^ and DS^–^ the favorable Coulomb interaction between the headgroups is much more important in enhancing the surfactant surface density than the favorable van der Waals interactions between the hydrophobic tails of DTA^+^ and DS^–^.

In summary, we performed HD-VSFG measurements on aqueous solutions of the negative surfactant SDS and the positive surfactant DTAB. We measured the response of the CH vibrations of the alkyl tails of the surfactants and the response of the OH stretch vibrations of water molecules close to the surface. We modeled the data with a modified Langmuir adsorption model in which we include the electrostatic energy of the surfactant ions and their counterions.

We find that a solution containing both SDS and DTAB has a much higher surface concentration of surfactant ions than those of solutions of the separate surfactants. Already for a solution containing 10 μM SDS and 10 μM DTAB a near-complete surface surfactant layer is formed, as evidenced from the CH response of the alkyl tails of the surfactants. These bulk concentrations are ∼1000 times lower than the critical micelle concentration of SDS and DTAB. Increasing the bulk concentration of DTAB from 10 to 200 μM for a solution containing 50 μM of SDS has surprisingly little effect on the CH response, indicating that the surface concentrations of DS^–^ and DTA^+^ hardly change. This strong enhancement of the surface concentration of DS^–^ and DTA^+^ is explained from the favorable Coulomb interaction of the oppositely charged headgroups of DS^–^ and DTA^+^.

For a solution containing 50 μM SDS, the response of the water OH vibrations strongly decreases when the bulk concentration of DTAB is increased from 10 to 200 μM, which can be explained by the change of the sign and amplitude of the surface potential. The water OH response is largely determined by the orienting effect of the surface potential. Calculations using the modified Langmuir adsorption model show that varying the bulk concentration of DTAB from 10 to 200 μM leads to a transition from a small excess of DS^–^ to a small excess of DTA^+^, which entails a sign change of the surface potential. For a solution containing the same concentration of SDS and DTAB, the surface concentrations of DS^–^ and DTA^+^ are the same, and the surface potential equals zero. Nevertheless, we observe a clear residual positive Im [χ^(2)^] signal associated with the water OH response, which can be explained from the contribution to the water OH response of water molecules that are directly interacting with the headgroups of DS^–^ and DTA^+^. The direct interaction between water OH groups with the negatively charged sulfate headgroup of DS^–^ has a stronger orienting effect than the direct interaction between water and the positively charged trimethylammonium headgroup of DTA^+^.

## Supporting Information Available

Additional experimental details: sample preparation and HD-VSFG technique including schematic representation of experimental setup, HD-VSFG spectra of binary mixture of 50 μM DAB and SDS/d_25_ SDS, HD-VSFG spectra of 50 μM SDS at various DTAB concentrations, modified Langmuir modeling and peak fitting (PDF)
Transparent Peer Review report available (PDF)
