# Supplementary material for: Observation of Electrostatically Driven Surface Adsorption in Mixed Surfactant Systems
Source: J Phys Chem Lett. 2024 Feb 2;15(6):1596–602. doi: 10.1021/acs.jpclett.3c03377 (PMC10875667; doi:10.1021/acs.jpclett.3c03377)
Supplement: Supplementary file 1 — jz3c03377_si_001.pdf [file jz3c03377_si_001.pdf]

Observation of Electrostatically Driven Surface Adsorption in Mixed Surfactant Systems

Aswathi Vilangottunjalil*, Jan Versluis, Huib J. Bakker

AMOLF, Ultrafast Spectroscopy, Science Park 104, Amsterdam, Netherlands

*a.vilangottunjalil@amolf.nl

Table of Contents

1. **Experimental Procedures** **2**
   1.1 Sample Preparation 2
   1.2 Heterodyne-Detected Vibrational Sum-Frequency Generation Spectroscopy 2
2. **Auxiliary Spectroscopic data** 4
   2.1 HD-VSFG spectra of spectra of binary mixture of 50 µM Dodecyl ammonium bromide (DAB) and Sodium dodecyl sulfate (SDS)/deuterated Sodium dodecyl sulfate (d_{25} SDS). 4
   2.2 HD-VSFG spectra of spectra of binary mixture of 50 µM deuterated Sodium dodecyl sulfate (d25 SDS) at various Dodecyl trimethyl ammonium (DTAB) concentration 5
3. **Modelling** 6
4. **Peak Fitting** 8
5. **References** 8

1. Experimental Procedures

1.1 Sample Preparation

Sodium dodecyl sulphate (SDS), dodecyl trimethyl ammonium bromide (DTAB) and Dodecyl ammonium bromide (DAB) (all purity ≥ 99.0 %) were purchased from Sigma. All surfactants were used as received without further purification. We made stock solutions of 10 mM SDS, 10 mM DTAB and 5mM DAB. Purified deionized water with a resistivity of 18.2 MΩ·cm was obtained from a Milli-Q system and used to prepare surfactant solutions in all experiments. A mixture of solutions of higher concentration i.e. 1 mM SDS + 1mM DTAB was murky, which indicates aggregation of the surfactants in the bulk solution. Therefore, we made solutions at lower concentrations, i.e. in the micromolar concentration regime, which are fully transparent. Solutions at lower concentrations were prepared by serial dilution from the stock solutions. We made separate solutions of SDS and DTAB and then mixed them (shaken well) at different ratios in separate vials to form the mixtures of different compositions. Subsequently, the solution is transferred to a Teflon trough and the measurement is done after a few minutes allowing the system to fully equilibrate. We varied the concentrations of SDS and DTAB from 10 – 200 µM. For the HD-VSFG measurements, 4 ml (about 0.14 oz) of the required solution was added to a in house-built round Teflon trough (4 cm diameter). The trough was cleaned several times with Millipore water and acetone and then dried with high purity nitrogen gas to avoid all possible contamination.

1.2 Heterodyne-Detected Vibrational Sum-Frequency Generation Spectroscopy

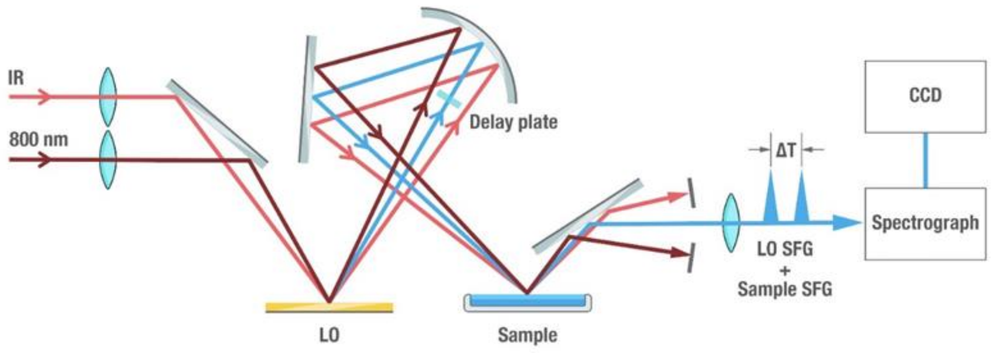

Figure S1. Schematic representation of the sample and detection compartments of the HD-VSFG setup.

The sum-frequency generation is enhanced for mid-infrared frequencies corresponding to vibrational resonances at the surface. HDVSFG is highly surface specific as the generation of sum-frequency light is symmetry forbidden in the bulk. The heterodyne detection enables us to determine both the real and the imaginary part of the second-order susceptibility. The sign of Im[χ(2)] carries information on the orientation of the probed vibrational transition dipole moment at the interface and hence provides direct information on the molecular structure at the interface.

All measurements were performed on a home-built HD-VSFG setup. It is based on a commercial Ti:Sapphire laser system, which consists of an oscillator (Coherent Mantis) which delivers 35 fs pulses centered at 800 nm and an regenerative amplifier (Coherent Legend). The resulting pulses have an energy of 3.5 mJ and a repetition rate of 1 kHz. The laser output is divided into two parts to generate a tunable broad band mid-infrared beam and a narrow 800 nm beam. Two thirds of the fundamental beam is used to pump a home-built OPA to produce the signal and idler which then later combined at the difference frequency generation DFG to generate infrared (ω_{IR}) centered at 3100 cm-1. The remaining one third is used to generate spectrally narrow 800 nm pulse (ω_{vis}) using an etalon. The energies of the ω_{IR} and ω_{vis} pulses are 15 μJ and 10 μJ, respectively. The broad-band mid-infrared pulse (ω_{IR}) and narrow band 800 nm (ω_{vis}) are focused and overlapped temporally and spatially on a gold

mirror which acts as a local oscillator (LO). This local oscillator SFG signal is then guided through a 1 mm silica plate to delay it in time with respect to other beams before all three beams (ω_{IR}, ω_{vis} , LO-SFG) are refocused on to the sample surface to generate sample VSFG signal. The LO SFG and sample SFG are sent into a spectrometer and the interference pattern of these two SFG beams are detected with an electron-multiplied charged coupled device (EMCCD). From the interference spectrum, the real (Re) and imaginary (Im) parts of $\chi^{(2)}$ spectra of the sample can be extracted, which contains the information about the molecular functional groups. We also performed a reference measurement in which the sample is replaced with quartz. We corrected the VSFG spectra for the spectral dependence of the input infrared beam by dividing the sample SFG signal with the reference SFG signal. To provide sufficient accuracy of the extracted phase, the quartz crystal was placed at the same height as the sample, which was controlled based on the vertical position of the signal on the CCD camera. Spectra were collected in SSP polarization combination (s-polarized SFG, s-polarized VIS, p-polarized IR).

2. Auxiliary Spectroscopic data

2.1 HD-VSFG spectra of spectra of binary mixture of 50 µM Dodecyl ammonium bromide (DAB) and Sodium dodecyl sulfate (SDS)/deuterated Sodium dodecyl sulfate (d_{25} SDS).

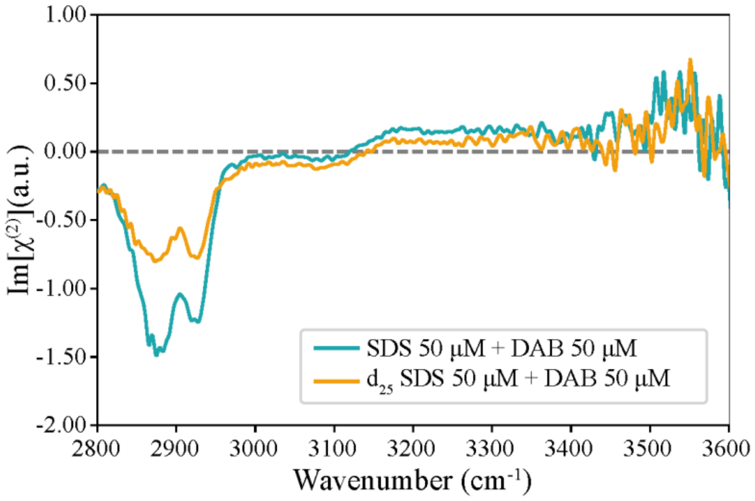

Figure S2. Im[$\chi^{(2)}$] spectra of aqueous solution 1:1 ratios of mixed surfactants of SDS and DAB water-air interface. For comparison the 1:1 ratio of 50 µM deuterated SDS and DAB.

We repeated the measurements with DAB instead of DTAB. DAB differs from DTAB in its head group; DTAB has three methyl groups whereas DAB has three hydrogens in the headgroup. In Figure S2 we observe that the residual water signal that was observed for 1:1 mixture of SDS and DTAB, vanishes for an equimolar solution of SDS and DAB. This finding implies that the positive water signal in Figure 2 of the main manuscript resulted from the more strongly orienting effect of the DS- head group compared to the DTA^+ headgroup, which is explained from the presence of three methyl groups in the headgroup of DTA^+. When these methyl groups are no longer there, as for DA^+, the positive and negative surfactants have equal competence in aligning the water molecules, resulting in a zero net water signal, as is observed in Figure S2.

2.2 HD-VSFG spectra of spectra of binary mixture of 50 µM deuterated Sodium dodecyl sulfate (d_{25} SDS) at various Dodecyl trimethyl ammonium (DTAB) concentration

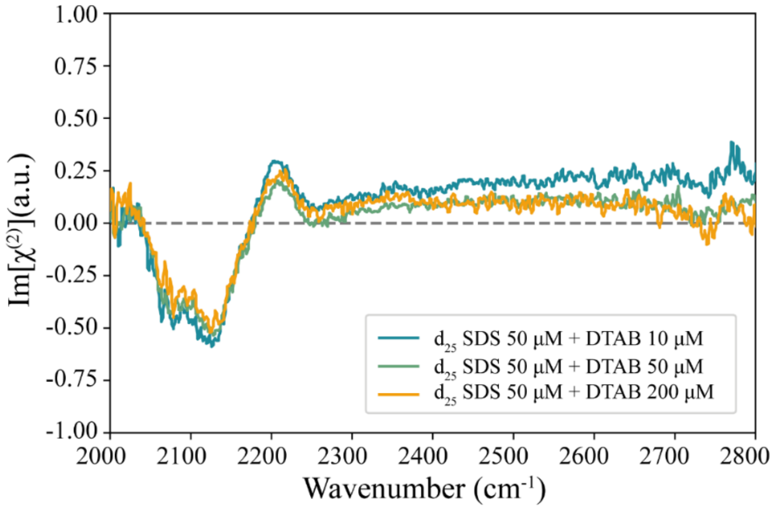

Figure S3. Im[$\chi^{(2)}$] spectra of aqueous solution of mixture surfactants of 50 µM deuterated SDS with different concentrations of DTAB ranging from 10 µM – 200 µM at the air-water interface examined in the C-D stretch region along with Im[$\chi^{(2)}$] spectra of D_2O.

We examined the CD and OD spectral region of the aqueous solutions of deuterated SDS 50 µM and different concentrations of DTAB to determine the surface occupancy of deuterated SDS. The isotopic substitution of the alkyl chain hydrogens by the heavier deuterium atoms in the SDS results in a red-shift of approximately 800 cm^{-1} in the spectrum. In Figure S3, we present the Im[$\chi^{(2)}$] spectra of D_2O (blue) as a reference. Since the solutions are made in H_2O, not in D_2O we do not see the OD stretches. However, in the CD region we observe two negative features at 2070 cm^{-1} and 2135 cm^{-1}, and a sharp positive peak at 2210 cm^{-1}. The 2070 cm^{-1} band is assigned to the symmetric CD stretch vibration of the terminal methyl, and the 2135 cm^{-1} band is assigned to the Fermi resonance of the symmetric CD stretch vibrations and the overtone of the CD bending mode of the CD_3 groups. The small positive peak at 2210 cm^{-1} is assigned to the antisymmetric CD stretch vibration of the terminal methyl group. These spectral assignments are consistent with a previous study of the Tyrode group[1] of deuterated hexane. From the amplitudes of the CD bands in the Im[$\chi^{(2)}$] spectra, it is clear that the surface occupancy of the SDS is the same in all three cases.

3. Modified Langmuir Modelling

In the Langmuir isotherm model[2] for a binary mixture, the surfactants (DS^- and DTA^+) compete to occupy the same surface sites for adsorption. This model assumes that the particles do not interact with each other, that the surface is uniform and homogeneous, and that a specific surface site cannot be simultaneously occupied by DS^- and DTA^+. To express the surface occupancy, θ of the separate surfactants, we can use the Langmuir isotherm equation:

$$\theta_{DS^-} = \frac{K_L^{sds} C_{sds}}{1 + K_L^{sds} C_{sds} + K_L^{dtab} C_{dtab}} \quad (1)$$

$$\theta_{DTA^+} = \frac{K_L^{dtab} C_{dtab}}{1 + K_L^{sds} C_{sds} + K_L^{dtab} C_{dtab}} \quad (2)$$

Where K_L term denotes the Langmuir constant of the surface adsorption, and the C term denotes the bulk concentration of the corresponding surfactant molecules,

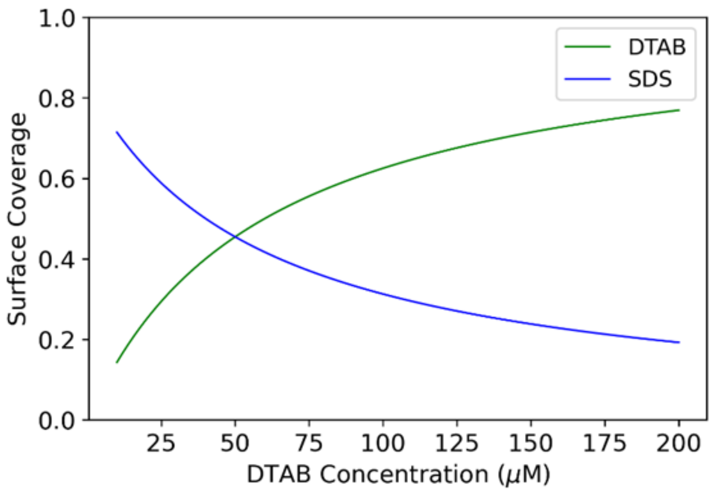

FigureS4. Surface coverage of SDS and DTAB calculated from standard Langmuir model with varying DTAB concentration and fixed SDS concentration of 50 μM.

The surface coverage of both the surfactants calculated using standard Langmuir adsorption model is depicted in Figure S4 with varying DTAB concentration keeping SDS concentration at 50 μM. Here the electrostatic interaction is not considered. The surface occupancy obtained from the Langmuir isotherm can be modified by taking the interaction of the surfactants into account. We calculated the surface occupancy based on the assumption that K_L^{sds} and K_L^{dtab} are similar which implies that both have similar surface propensity.

4. Peak Fitting

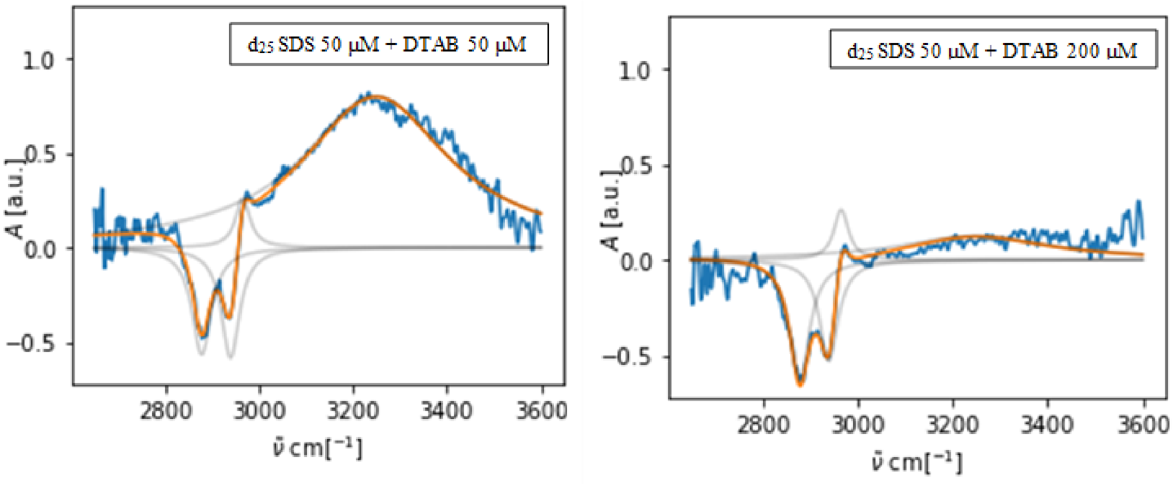

Figure S5. The Im[$\chi^{(2)}$]spectra of d_{25} SDS 50 µM + DTAB 50 µM and d_{25} SDS 50 µM + DTAB 200 µM. The measured data are represented by the blue and the fitted spectra by orange lines.

We fitted the spectra to a set of Lorentzian by minimizing the sum of least squares of the difference between a set of mixture of surfactants SFG spectra and a set of Lorentzian functions. The detailed explanation for the fitting is explained elsewhere[4]. The Im[χ(2)] spectra can be well decomposed into four Lorentzian bands centered at 2878 cm^{-1}, 2939 cm^{-1}, 2965 cm^{-1} and 3248 cm^{-1}. In the fitting we kept the widths of all Lorentzian bands the same at all DTAB concentrations and only allowed the amplitudes of the bands to change in dependence on the DTAB concentration.

References

(1) Tyrode, E.; Hedberg, J. A Comparative Study of the CD and CH Stretching Spectral Regions of Typical Surfactants Systems Using VSFS: Orientation Analysis of the Terminal CH3 and CD3 Groups. *J. Phys. Chem. C* **2012**, *116* (1), 1080–1091.

(2) Ertl, G. Reactions at solid surfaces, John Wiley & Sons, **2010**.

(3) Hans-Jürgen Butt, Karlheinz Graf, M. K. Physics and Chemistry of Interfaces; John Wiley & Sons, **2023**.

(4) Antalicz, B.; Sengupta S.; Vilangottunjalil A.; Versluis J.; and Bakker, H. J. Orientational Behavior and Vibrational Response of Glycine at Aqueous Interfaces, J. Phys. Chem. Lett. 2024, (in press)
