# Supplementary material for: Observation of Electrostatically Driven Surface Adsorption in Mixed Surfactant Systems
Source: J Phys Chem Lett. 2024 Feb 2;15(6):1596–602. doi: 10.1021/acs.jpclett.3c03377 (PMC10875667; doi:10.1021/acs.jpclett.3c03377)
Supplement: Supplementary file 2 — jz3c03377_si_002.pdf [file jz3c03377_si_002.pdf]

jz-2023-033774.R1

Name: Peer Review Information for "Observation of Electrostatically Driven Surface Adsorption in Mixed Surfactant Systems"

First Round of Reviewer Comments

Reviewer: 1

Comments to the Author

This submitted work entitled "Observation of Electrostatically Driven Surface Adsorption in Mixed Surfactant Systems" by Bakker et al. primarily used heterodyne detected SFG to elucidate the molecular interaction of SDS and DTAB adsorbed layer at the air/water interface.

This enhancement in the adsorption of oppositely charged surfactants has been known and reported long ago; thus this study is not new in this regard. In addition, I find this work not ready to be considered in JPCL due to this major flaw: the adsorption of surfactant at the air/water interface occurs very fast (mostly instantly); thus the subsequent introduction of small amounts of surfactants after the initial adsorption layer has formed would not be the same as introducing the large amount surfactant at the beginning, especially in the cases when the surfactant excess at the interface is large (as in this study). The reason behind is the alkyl chains hydrophobic interaction among the surfactant molecule is rather rigid, once the adsorbed layer is formed, the energy barrier to break it is high.

I have a feeling that some of the authors writing this manuscript is new to SFG; a few statements here and there are conceptually incorrect. For example, the statement at the bottom of pg9 when they said a 50% drop in the signal detected would infer a 50% drop in number density. This is wrong as sfg signal is squared of number density.

The TOC was incorrectly plotted as the adsorption layer of DTAB and SDS at the air/water interface would be very dense (the authors can construct the isotherm of the adsorption using surface tension measurements to see this), we expect the alkyl chains of the surfactant species to intimately interact with each other. Also, did the authors observe surfactant aggregation (or the solution becomes murky) when mixing DTAB and SDS together in one pot? as upon the electrostatic force is dismissed the solubility of the surfactant species drops dramatically.

I suggest the authors revise this manuscript carefully and submit it to another JPC journal.

Reviewer: 2

Comments to the Author

The interaction of surfactants with their environments and themselves is relevant in fields such as biology, environmental science, and fundamental physical chemistry. Due to the hydrophilic head group and hydrophobic tail, the behavior at hydrophobic/hydrophilic interfaces, e.g., air/water interface, can be extremely sensitive to Coulomb interactions and the solubility of the molecules. In this contribution, Huib and co-workers conducted a comprehensive study of the interaction of two surfactants, SDS (negative), and DTAB (positive), in aqueous solutions using HD-VSFG measurements. They found that the coexistence of SDS and DTAB in a solution leads to a significantly higher surface concentration of surfactant ions compared to individual solutions. Increasing DTAB bulk concentration from 10 to 200 μM in a solution containing 50 μM SDS has minimal impact on the surface concentration. The response of water OH vibrations experiences a pronounced decrease with increased DTAB concentration. There is some direct interaction of water molecules with the sulfate headgroup of DS-, causing a stronger orienting effect than with the trimethylammonium headgroup of DTA+. This study provides valuable insights into the intricate interplay between surfactants and water molecules at the solution interface. The observed effects on surface concentrations and potential, particularly in mixed solutions, highlight the significance of Coulomb interactions and direct interactions in shaping interfacial properties. These findings contribute to a deeper understanding of surfactant behavior and have potential implications for various applications in colloid and surface science. The manuscript has been well-written and should be published after considering my following comments.

While adding the Coulomb interaction term to the Langmuir isotherm works well in explaining the results, the underlying physics should be elaborated, and the new equation should be justified either by deriving from adsorption reaction equations or providing specific references.

The abstract should be extended to include the main findings of this study rather than just generally interpreting the methods.

The expression "Gibbs free energy of the electric double layer" should be modified, as DEL does not have net Gibbs free energy.

On page 22, the discussion of the difference between C12E6 and DTAB should either be cut or extended to compare the quantitative differences. Obviously, DTA and DS also have strong Van der Waals interactions.

A few typos: on page 15, line 35, it should read "For low potentials, ΔG can..."; on page 17, line 43-44, it should read "... that in turn is governed by the..."; on page 20, lines 36-37, it should read "by Kawai et al., show mixed..."; on page 22, lines 23-24, "Van der Waals."

Author's Response to Peer Review Comments:

Dear Editor,

We hereby would like to resubmit our manuscript jz-2023-033774, titled “Observation of Electrostatically Driven Surface Adsorption in Mixed Surfactant Systems”.

We would like to thank you for your handling of our manuscript and the reviewers for their valuable comments and suggestions for improving our manuscript. We have carefully revised the paper according to the recommendations of the reviewers. Below you will find detailed replies to the comments of the reviewers, in which we also explain how we changed the manuscript accordingly.

We hope that the revised manuscript will be suitable for publication in the Journal of Physical Chemistry Letters.

With kind regards,

Aswathi Vilangottunjalil, Jan Versluis, Huib J. Bakker

Editor's requests

Request 1:

Supporting Information Statement: A brief, non-sentence description of the actual contents of each supporting information file is required. This description should be labeled Supporting Information and should appear before the Acknowledgement and Reference sections. Examples of sufficient and insufficient descriptions are as follows:
*Examples of sufficient descriptions: "Supporting Information: 1H NMR spectra for all compounds" or "Additional experimental details, materials, and methods, including photographs of experimental setup".
*Examples of insufficient descriptions: "Supporting Information: Figures S1-S3" or "Additional figures as mentioned in the text".

Response:

We now added a separate section labeled as "Supporting information" to the manuscript in which we describe the actual contents of the supporting information.

Request 2:

Supporting Information: Please add full header at top of page of the Supporting Information file, which includes: Title, Full Author List, and Author affiliations (exactly as they appear in the manuscript).

Response:

We added a full header at the top page of the supporting information file, including the title, author list and author affiliations.

Request 3:

TOC Graphic: Please resize the TOC graphic per journal guidelines (2 in x 2 in)

Response:

We updated the TOC figure to fully comply with the journal-specific guidelines.

Request 4:

References: In both the main file and the supporting information, fix the style of all references to use JPCL formatting (check all references carefully). ***JPC Letters reference formatting requires that journal references should contain: () around numbers; author names; article title (titles entirely in title case or entirely in lower case); abbreviated journal title (italicized); year (bolded); volume (italicized); and pages (first-last). Book references should contain author names; book title (in the same pattern); publisher; city; and year. Websites must include date of access

Response:

We carefully checked and updated each reference, in both the main text and the SI; and used the appropriate abbreviations for all the journal names. Additionally, we also updated the reference formatting via the ACS Latex style template.

Request 5:

References: URLs are not preferred references because website content can be modified and, consequently, the reference information may lack permanence.

Response:

We removed all the URLs from the reference section and updated the reference section according to the journal guidelines.

Request 6:

Supporting Information: Please number SI pages in the following format: “S1, S2…”

Response:

We corrected the Supplementary information page numbering.

Reviewer: 1

Comment 1:

This submitted work entitled "Observation of Electrostatically Driven Surface Adsorption in Mixed Surfactant Systems" by Bakker et al. primarily used heterodyne detected SFG to elucidate the molecular interaction of SDS and DTAB adsorbed layer at the air/water interface.

This enhancement in the adsorption of oppositely charged surfactants has been known and reported long ago; thus this study is not new in this regard. In addition, I find this work not ready to be considered in JPCL due to this major flaw: the adsorption of surfactant at the air/water interface occurs very fast (mostly instantly); thus the subsequent introduction of small amounts of surfactants after the initial adsorption layer has formed would not be the same as introducing the large amount surfactant at the beginning, especially in the cases when the surfactant excess at the interface is large (as in this study). The reason behind is the alkyl chains hydrophobic interaction among the surfactant molecule is rather rigid, once the adsorbed layer is formed, the energy barrier to break it is high.

Authors reply:

We thank the reviewer for carefully reading our manuscript and for the very useful comments and suggestions. We think that there exists an unfortunate misunderstanding in how the samples are prepared. We did not add small amounts of additional surfactants to the sample having already an initial adsorption layer. Instead, we made separate solutions of SDS and DTAB and then mixed them (shaken well) at different ratios in separate vials to form the mixtures of different compositions. Subsequently, each solution is transferred to a Teflon trough and the measurement is done after few minutes, allowing the system to fully equilibrate and to form a Gibbs layer in thermodynamic equilibrium.

Action Taken:

We now included the additional text to the sample preparation part of supplementary information page S2.

"We made separate solutions of SDS and DTAB and then mixed them (shaken well) at different ratios in separate vials to form the mixtures of different compositions. Subsequently, the solution is transferred to a Teflon trough and the measurement is done after a few minutes allowing the system to fully equilibrate."

Comment 2:

I have a feeling that some of the authors writing this manuscript is new to SFG; a few statements here and there are conceptually incorrect. For example, the statement at the bottom of pg9 when they said a 50% drop in the signal detected would infer a 50% drop in number density. This is wrong as sfg signal is squared of number density.

Authors reply:

We thank the reviewer for noting this mistake. Indeed, there was a miswording in Page 9, line 44-45, where we used the wording "CH signal intensity" instead of "CH signal amplitude". As the Reviewer correctly points out, conventional (homodyne detected) SFG measures the sum-frequency light intensity which scales quadratically with the surface number density. In our heterodyne detected vibration sum-frequency generation (HD-VSFG) experiments, the signal scales linearly with the number density, hence in this case a 50% drop in signal detected indeed implies a 50% drop in number density.

(*Stiopkin, I. V., Jayathilake, H. D., Bordenyuk, A. N., & Benderskii, A. V. (2008). Heterodyne-detected vibrational sum frequency generation spectroscopy. Journal of the American Chemical Society, 130(7), 2271-2275.)*

The above-mentioned reference illustrates the scaling of the homodyne-detected SFG intensity and HD-SFG signal amplitude of the CH_3 symmetric stretch mode with the surface coverage (1-octanol mol fraction in the monolayer).

Action Taken:

We have replaced the word 'CH signal intensity' to 'CH signal amplitude' on page 9.

Comment 3:

The TOC was incorrectly plotted as the adsorption layer of DTAB and SDS at the

air/water interface would be very dense (the authors can construct the isotherm of the adsorption using surface tension measurements to see this), we expect the alkyl chains of the surfactant species to intimately interact with each other.

Authors reply:

We thank the reviewer for this useful suggestion, and we updated the TOC.

Action Taken:

We modified the TOC as suggested by the reviewer. We added more surfactant molecules at the air-water interface to illustrate that the adsorption layer is densely packed.

Comment 4:

Also, did the authors observe surfactant aggregation (or the solution becomes murky) when mixing DTAB and SDS together in one pot? as upon the electrostatic force is dismissed the solubility of the surfactant species drops dramatically.

Authors reply:

We initially tried to make solutions of 1mM SDS + 1mM DTAB, and indeed the solution was murky and forming aggregates, we then prepared solutions at much lower concentrations, i.e. in the micromolar range. The obtained solutions were completely transparent.

Action Taken:

We now included the additional text to the sample preparation part of supplementary information.

"A mixture of solutions of higher concentration i.e. 1 mM SDS + 1mM DTAB was murky, which indicates aggregation of the surfactants in the bulk solution. Therefore, we made solutions at lower concentrations, i.e. in the micromolar concentration

regime, which are fully transparent. Solutions at lower concentrations were prepared by serial dilution from the stock solutions."

Reviewer: 2

Comment 1:

The interaction of surfactants with their environments and themselves is relevant in fields such as biology, environmental science, and fundamental physical chemistry. Due to the hydrophilic head group and hydrophobic tail, the behavior at hydrophobic/hydrophilic interfaces, e.g., air/water interface, can be extremely sensitive to Coulomb interactions and the solubility of the molecules. In this contribution, Huib and co-workers conducted a comprehensive study of the interaction of two surfactants, SDS (negative), and DTAB (positive), in aqueous solutions using HD-VSFG measurements. They found that the coexistence of SDS and DTAB in a solution leads to a significantly higher surface concentration of surfactant ions compared to individual solutions. Increasing DTAB bulk concentration from 10 to 200 μM in a solution containing 50 μM SDS has minimal impact on the surface concentration. The response of water OH vibrations experiences a pronounced decrease with increased DTAB concentration. There is some direct interaction of water molecules with the sulfate headgroup of DS-, causing a stronger orienting effect than with the trimethylammonium headgroup of DTA+. This study provides valuable insights into the intricate interplay between surfactants and water molecules at the solution interface. The observed effects on surface concentrations and potential, particularly in mixed solutions, highlight the significance of Coulomb interactions and direct interactions in shaping interfacial properties. These findings contribute to a deeper understanding of surfactant behavior and have potential

implications for various applications in colloid and surface science. The manuscript has been well-written and should be published after considering my following comments.

Authors reply:

We thank the reviewer for carefully reading our manuscript and for the very useful comments and suggestions.

Comment 2:

While adding the Coulomb interaction term to the Langmuir isotherm works well in explaining the results, the underlying physics should be elaborated, and the new equation should be justified either by deriving from adsorption reaction equations or providing specific references.

Authors reply:

We thank the reviewer for this valuable comment, and we modified and elaborated the modelling part to provide a clearer physical picture. We also added a few appropriate references.

Action Taken:

We revised the following text in the manuscript:

The surface occupancy obtained with the Langmuir adsorption model is:

$$\theta_{DS^-} = \frac{K_L^{sds} C_{sds}}{1 + K_L^{sds} C_{sds} + K_L^{dtab} C_{dtab}} \quad (1)$$

$$\theta_{DTA^+} = \frac{K_L^{dtab} C_{dtab}}{1 + K_L^{sds} C_{sds} + K_L^{dtab} C_{dtab}} \quad (2)$$

The K_L terms represent Langmuir equilibrium adsorption constants which can be expressed in terms of Gibbs free energy[33], and the C terms represent the bulk concentrations.

$$K_L = e^{-\frac{\Delta G}{k_b T}} \qquad (3)$$

Where ΔG is the total Gibbs free energy change associated with adsorption to the surface, k_b is Boltzmann's constant, and T is the temperature. For the case of a charged surfactant like DS^- and DTA^+, the value of ΔG and thus K_L depends on electrostatic interactions governed by the ionic strength and the presence of other charged surfactants and their counterions. ΔG can thus be separated in a non-electrostatic part ΔG_{nel} which accounts for the standard Gibbs energy of adsorption and an electrostatic part ΔG_{el} :

$$\Delta G = \Delta G_{nel} + \Delta G_{el} \qquad (4)$$

The electrostatic energy term is composed of the energy associated with adsorption of the surfactant ions at the surface and that of their counterions in the diffuse double layer,

$$\Delta G_{el} = \Delta G_{si} + \Delta G_{ci} \qquad (5)$$

The counterions are distributed over a region that extends some distance from the surface into the bulk because of the thermal motion of counterions. Hence, the energy associated with the counterions can be determined by integrating over the depth of the double layer as shown in equation 6.

$$\Delta G_{ci} = \frac{e\int_0^{\infty} \phi(z)\rho(z)dz}{\int_0^{\infty} \rho(z)dz} \qquad (6)$$

Where ρ(z) is the charge density and ϕ(z) is the electrostatic potential at position z. The electrostatic energy of the surfactant ions at the surface is $-e\phi_0$. Solving the Poisson Boltzmann equation for the potential ϕ(z), using the low potential assumption[33], we obtain for ΔG_{el}

$$\Delta G_{el} \approx -k_bT\, sinh^{-1}(\frac{\sigma}{\sqrt{8C\varepsilon\varepsilon_0 k_bT}}) \qquad (7)$$

where σ is the surface charge density that depends on the surface occupancy and the area a occupied by each surfactant obtained from the Langmuir isotherm (σ = e(θ_{DTA^+} -θ_{DS^-})/a), C is the total concentration of monovalent salts (C_{sds} + C_{dtab}) in mol/l, and ε is the static permittivity of the solution. We solve equations (1), (2), (3), (4), and (7) self-consistently for θ_{DTA^+} and θ_{DS^-} for each mixture with concentrations C_{sds} and C_{dtab}.

Comment 3:

The abstract should be extended to include the main findings of this study rather than just generally interpreting the methods.

Authors reply:

We thank the reviewer for pointing this out. We modified the abstract accordingly.

Action Taken:

We added the following to the abstract.

"*We observed that these surfactants show a strong cooperative effect in their adsorption to the water-air interface. Even at bulk concentrations 1000 times lower than the critical micelle concentration of SDS and DTAB, a nearly complete surface surfactant layer is observed when both surfactants are present. This strong enhancement of the surface concentration of DS^- and DTA^+ can be quantitatively explained from the favorable Coulomb interaction of the oppositely charged headgroups of DS^- and DTA^+ and the electrostatic interactions with their counterions.*"

Comment 4:

The expression "Gibbs free energy of the electric double layer" should be modified, as DEL does not have net Gibbs free energy.

Authors reply:

Following the suggestion of the Reviewer, we strongly revised the description of how we model the interactions of the surfactants and their counterions. We corrected the expression.

Action Taken:

We changed it to “Gibbs free energy change associated with adsorption to the surface”

Comment 5:

On page 22, the discussion of the difference between C12E6 and DTAB should either be cut or extended to compare the quantitative differences. Obviously, DTA and DS also have strong Van der Waals interactions.

Authors reply:

We agree with the reviewer that the DTA^+ and DS^- ions will also have strong Van der Waals interactions. The description in the original manuscript was indeed incomplete.

Action Taken:

We also added the following to the text in Page 22, line 24-25

“In this case, the enhancement results from the favorable Van der Waals interaction of the alkyl tails of the $C_{12}E_6$ and DS^- surfactants which is in fact also present in the case of DTA^+ and DS^-. However, in the case of DTA^+ and DS^- the favorable coulomb interaction between the headgroups is much more important in enhancing the

surfactant surface density than the favorable Van der Waals interactions between the hydrophobic tails of DTA^+ and DS^-."

Comment 6:

A few typos: on page 15, line 35, it should read "For low potentials, ΔG can…"; on page 17, line 43-44, it should read "… that in turn is governed by the…"; on page 20, lines 36-37, it should read "by Kawai et al., show mixed…"; on page 22, lines 23-24, "Van der Waals."

Authors reply:

We thank the reviewer for pointing out these typos.

Action Taken:

All typos fixed.
